# A dataset of distribution and diversity of ticks in China

**DOI:** 10.1038/s41597-019-0115-5

**Published:** 2019-07-01

**Authors:** Guanshi Zhang, Duo Zheng, Yuqin Tian, Sen Li

**Affiliations:** 1 School of Environmental Science and Engineering, Huazhong University of Science and Technology, Wuhan, P.R. China; 2 Centre for Ecology & Hydrology, Wallingford, UK; 3 Environmental Change Institute, University of Oxford, Oxford, UK

**Keywords:** Ecological epidemiology, Biogeography, Risk factors, Entomology

## Abstract

While tick-borne zoonoses, such as Lyme disease and tick-borne encephalitis, present an increasing global concern, knowledge of their vectors’ distribution remains limited, especially for China. In this paper, we present the first comprehensive dataset of known tick species and their distributions in China, derived from peer-reviewed literature published between 1960 and 2017. We searched for journal articles, conference papers and degree thesis published in both English and Chinese, extracted geographic information associated with tick occurrence, and applied quality-control procedures to remove duplicates and ensure accuracy. The dataset contains 5731 records of geo-referenced occurrences for 123 tick species distributed over 1141 locations distinguished at four levels of scale i.e., provincial, prefectural, county, and township and finer. The most frequently reported tick species include *Haemaphysalis longicornis*, *Dermacentor silvarum*, *Ixodes persulcatus*, *Haemaphysalis conicinna*, *Rhipicephalus microplus*, and *Rhipicephalus sanguineus* sensu lato. The geographical dataset provides an improved map of where ticks inhabit China and can be used for a variety of spatial analyses of ticks and the risk of zoonoses they transmit.

## Abstract (summary)

**Table:** 

Design Type(s)	disease state design • data integration objective • pathogenicity design
Measurement Type(s)	Scientific Publication
Technology Type(s)	digital curation
Factor Type(s)	geographic location • temporal_interval • Species
Sample Characteristic(s)	Ixodida • China

Machine-accessible metadata file describing the reported data (ISA-Tab format)

## Background & Summary

Ticks are parasites distributed widely across the world. They are vectors of pathogens of many human and animal infectious diseases of global importance, including Lyme disease (LD), tick-borne encephalitis, haemorrhagic fever, tick-borne macular fever, Q-fever, Babesiosis, tick paralysis, *etc*.^1–3^. Approximately 10% of the currently known 867 tick species worldwide are reported as vectors of these pathogens^4,5^. However, vaccines against tick-borne diseases remain largely unavailable, especially for LD which is the most prevalent vector-borne disease in the northern temperate zone^6,7^. As a result, there is growing interest in understanding the distribution of tick species to better manage the transmission risk of the pathogens they carry^8^.

China is a vast country with a diversity of ecosystems and climatic zones which make many areas suitable for tick survival. To address devastating environmental crises, China has been advancing policies to protect biodiversity and restore forest ecosystem^9^ which may favour tick survival^10^. Moreover, domestic eco-tourism has been expanding rapidly^11^, implying more people may spend time in places ticks inhabit and, thus, a rising risk human-tick contact. According to Wu *et al*.^12^, 119 species of ticks have been found in China by 2013, accounting for about 13.7% of the total tick species identified over the world. At present, the literature on ticks in China mainly focuses on reporting the notification of ticks species in particular locations and/or tick-borne pathogens, however, studies providing a comprehensive and systematic description of geographic distribution and diversity of tick species are rare, with Wu *et al*.^12^ and Chen *et al*.^13^ being notable exceptions. As both of these studies were published five years ago (i.e. in 2010 and 2013, respectively) and focused on a coarse province-level, there is a need to update this information with most up-to-date records and their geo-locations at finer geographic scales. Furthermore, as tick-related records were better documented in Chinese, a study breaking language barriers for a more complete picture of tick-infested landscapes in China would be useful for future disease risk analysis and modelling experiments.

The dataset described here comprises 5731 records of geo-referenced tick occurrence reported from 1960 to 2017, concerning 123 species of ticks in 1141 locations across China. The most frequently reported tick species are *Haemaphysalis longicornis*, *Dermacentor silvarum*, *Ixodes persulcatus*, *Haemaphysalis conicinna*, *Rhipicephalus microplus*, and *Rhipicephalus sanguineus* sensu lato.

## Methods

### Data collection

**Fig. 1:**
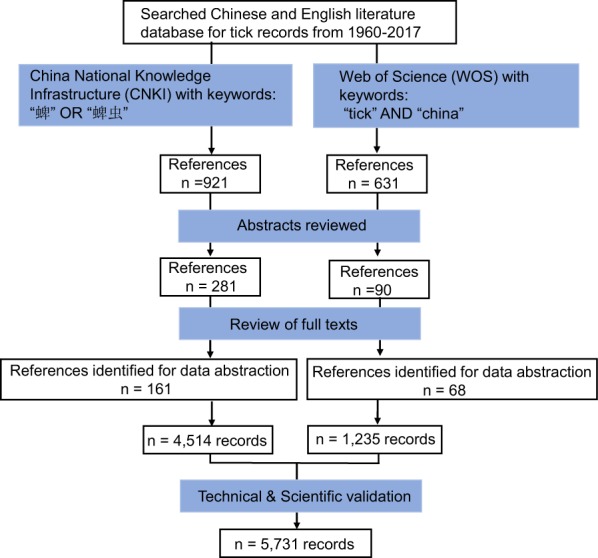
Schematic overview of the literature search procedure and results.

Our procedures of literature review are outlined in Fig. 1. Publications in both Chinese and English were collected by searching the two major scientific citation indexing services, the Web of Science (WOS) (http://apps.webofknowledge.com/) and China National Knowledge Infrastructure (CNKI) (http://www.cnki.net/), respectively. PubMed was not used because it largely overlaps with WOS and CNKI. In particular it contains many English abstracts of Chinese literature that are covered already in CNKI. As we focused on a time period from 1960 to 2017, the searches were last updated on 18^th^ August 2018 to ensure all literature published in 2017 is indexed. We used the terms (‘tick’ AND ‘China’) with WOS, and (“蜱” OR “蜱虫”) (both mean tick) with CNKI. A publication (a journal article, conference proceeding or degree thesis) was retrieved if the terms appeared in any parts of its content. No language restrictions were placed on these searches.

**Fig. 2:**
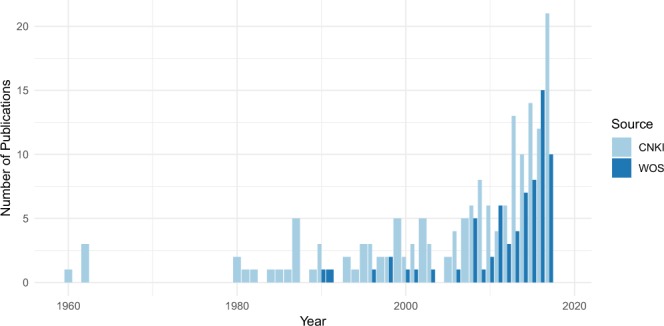
Increasing numbers of publications of tick occurrence acquired from the two sources (CNKI and WOS).

A total of 1552 abstracts were retrieved for screening, of which 921 were from CNKI (in Chinese) and 621 from WOS (in English). Abstracts which report only laboratory findings, describe identification of tick-borne diseases or do not include any geographic information were excluded. This led to 281 Chinese and 90 English papers being selected for full-text review and further extraction of geo-information. Then, through requesting and intensive reading of the available full-texts, 161 Chinese and 68 English publications were identified to be eligible for extraction. The earliest Chinese and English publications were published in 1960 and 1990, respectively. The number of publications of tick occurrence is increasing in recent years, suggesting a growing scientific interest (Fig. 2). A full list of publications reviewed is provided in the online dataset^14^.

The key information extracted from the literature include: (i) name of the tick species, (ii) location associated with tick occurrence (and its geographic scale), (iii) time of tick collection, and (iv) collection methods (e.g., drag/flag sampling, collecting from host, literature review, etc.). After the data were entered, a second person checked the dataset thoroughly to avoid errors and duplications. It is very common that a publication reports several tick species at different locations, and these were separated so that each record in our dataset represents an occurrence of a tick species in a location reported in specific year by an author. Finally, the dataset was re-evaluated to include historical changes in tick taxonomy and validity of novel observations. As a result, 5731 records of tick occurrence were complied, of which 4498 records were from CNKI and 1233 from WOS.

### Geo-positioning

Location information was extracted for each record from the relevant primary paper. As many publications reported only the administrative regions of tick occurrence, and a tick-infested location was likely to be reported in different publications, the records of location are duplicated to a certain extent. We thus aggregated location records first to reduce repeated geo-positioning operations which could induce redundancy and error. In total 1141 locations were identified. Similar to Kraemer *et al*.^15^, longitude and latitude of a location were determined using a combination of geospatial tools, including the xGeocoding software (http://www.gpsspg.com/xgeocoding/, with APIs to access georeference functions of the most commonly used online map services in China, namely, Baidu Map, Tecent’s QQ Map and Amap), Google Earth (http://www.google.co.uk/intl/en_uk/earth), or as a last resort, using simple keyword searches with Google or Baidu. Latitude and longitude are usually the centre of an administrative region, unless a particular place was specified, or coordinates were provided in the primary paper. We updated places’ names to match historical administrative names. We further classified all the locations into four different levels according to their geographic scales and administrative levels (i.e. provincial, prefectural, county, and township and finer level). This helps potential users of this dataset extract proper sections to use. The locations of tick occurrence were then visualised using geographic information systems (GIS) software. The administrative boundary maps (2015) used for displaying the results were retrieved from the Resource and Environment Data Cloud Platform, Chinese Academy of Sciences (http://www.resdc.cn/data.aspx?DATAID=202).

## Data Records

In the dataset of distribution and diversity of ticks in China (available from figshare^14^), each of the rows represents a single record (an occurrence of tick species in a location as reported in specific year by a reference). The columns contained in the dataset are as follows:

1. **tick_sp:** Identifying the species of ticks.
2. **lon:** The longitudinal coordinate of the location of tick occurrence (WGS1984 Datum).
3. **lat:** The latitudinal coordinate of the location of tick occurrence (WGS1984 Datum).
4. **loc_level:** the geographic scale of location (1 = provincial level, 2 = prefectural level, 3 = county level, 4 = township or finer level).
5. **loc_l1:** provincial level information of the location (name of province, autonomous region, municipality, or special administrative region of China).
6. **loc_l2:** prefectural level information of the location (name of prefectural-level city, or autonomous prefecture).
7. **loc_l3:** county level information of the location (name of county-level city, autonomous banner, district or county).
8. **loc_l4:** township or finer level information of the location.
9. **smp_methods:** sampling/collection methods (whether the tick records were acquired via dragging, flagging or trapping in the field, collected from host, or summarised by literature review, questionnaire or expert knowledge).
10. **smp_stt:** start year of tick sampling/collection.
11. **smp_end:** end year of tick sampling/collection.
12. **pub_t:** the year of the publication.
13. **pub_id:** identification number of references (those start with letters ‘c’ and ‘w’ indicate that the reference is retrieved from CNKI and WOS, respectively).
14. **pub_full:** references identified for data extraction.

## Technical Validation

There are 5731 records of tick occurrence extracted from literature published between 1960 and 2017. All records were initially extracted by one team member and then confirmed by another member. While at the stage of geo-positioning, a third person was involved so that data were checked again. Data were checked strictly to ensure accuracy and extraction criteria were met, similar to the approach used in Battle *et al*.^16^.

In this study, there were three situations wherein information from Chinese literature needs examinations before entering into the dataset. Firstly, tick species that were given different names in Chinese appear to have referred to identical scientific names using binomial nomenclature. Secondly, errors and typos in ticks’ scientific names were noted. Thirdly, inconsistency in the identification of tick species was found in the same article. In most cases, corrections could be done directly according to the context, morphological characteristics (as described in text or observed form the figures therein) and information extracted from other papers. The world list of ticks^17^ and the taxonomic literature of ticks in China^13^ were used as key references. Species whose names could not be confirmed were excluded.

**Fig. 3:**
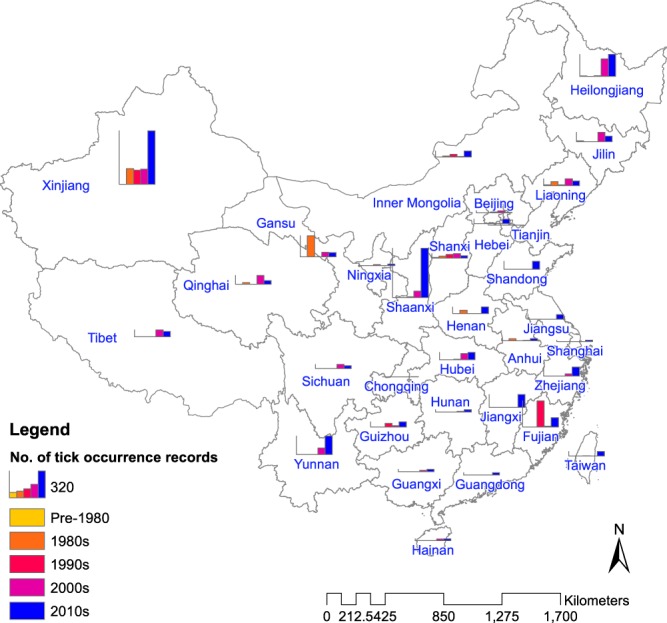
Number of tick occurrence records by time period across provincial-level divisions of China.

**Fig. 4:**
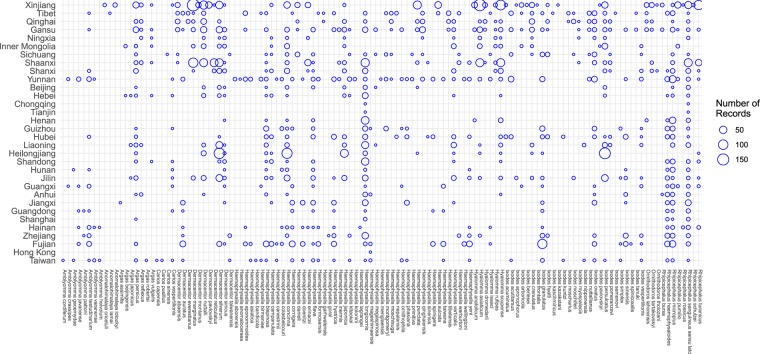
Number of records for different tick species by provincial-level division of China.

**Fig. 5:**
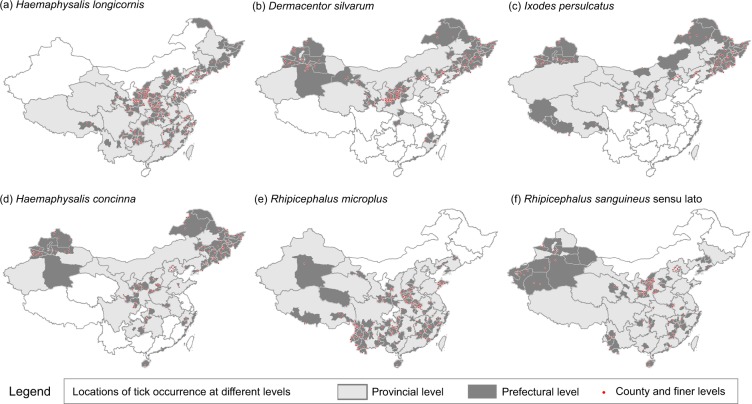
Locations of tick occurrence of six most frequently reported tick species in China. (**a**) *Haemaphysalis longicornis*. (**b**) *Dermacentor silvarum*. (**c**) *Ixodes persulcatus*. (**d**) *Haemaphysalis concinna*. (**e**) *Rhipicephalus microplus*. (**f**) *Rhipicephalus sanguineus sensu lato*.

It is important to ensure that locations of tick occurrence were duly georeferenced. Sometimes, locations were described incompletely, and, hence, difficult to be geo-positioned through the geospatial tools mentioned previously. For example, some locations were reported using abbreviation or ethnic languages of China. Some others only contained places on a very fine level in rural China (e.g. hill names in village) which could not be identified via any online search services. Occasionally, places may have changed their names from when the research was carried out. These all required intensive reading of the primary article, repeated checking with Google/Baidu, and analysing the semantics obtained from different sources. It thus makes it necessary to include the ‘loc_level’ field in the dataset, so that the readers are aware of our confidence in the spatial precision of each record. Finally, coordinates extracted by xGeocoding were mapped using Google Earth to ensure each location was pinned into the correct administrative regions in China. The resulting locations of tick occurrence as depicted in Figs 3–5 agree well with the previous findings and maps^18,19^.

Given the long span of the literature search (between 1960 and 2017), historical changes in prior taxonomic classifications need to be captured. After a thorough re-evaluation, following synonymisations were considered: *Dermacentor niveus* and *Dermacentor daghestanicus* as synonyms of *Dermacentor marginatus*^20^; *Dermacentor abaensis* as synonym of *Dermacentor everestianus*^21^; *Haemaphysalis vietnamensis* as synonym of *Haemaphysalis colasbelcouri*^22^, *Hyalomma detritum* as synonym of *Hyalomma scupense*^23^; *Ixodes rangtangensis* as synonym of *Ixodes moschiferi*^23^; *Ixodes redikorzevi* as synonym of *Ixodes acuminatus* (following assumption by Estrada-Pena *et al*.^20^); *Ixodes ochotonarius* as synonym of *Ixodes hyatti*^24^.

We further compared our records with recent scientific updates on tick fauna in China by Chen *et al*.^13^ and Guo *et al*.^25^, and included following species:

- (+) *Argas reflexus*, which is widely distributed in the Western Palaearctic, was reported in several northern provinces, including Xinjiang, Qinghai, Ningxia and Gansu. Its molecular identification is provided in a study by Zhao *et al*.^26^.
- (+) *Anomalohimalaya cricetuli* was reported several times in Xinjiang and suggested as a valid species name by Guglielmone *et al*.^27^.
- (+) *Haemaphysalis inermis* was reported in several provinces in China, including Gansu, Shaanxi and Yunnan. Nosek^28^ described *Ha. inermis* as of South Asia origin. Robbins and Robbins^24^ examined misidentification of several early Chinese and Japanese tick studies and reported that Teng (1983) has collected *Ha. inermis* from Sichuan. Chitimia-Dobler *et al*.^29^ explains that the distribution of *Ha. inermis, Haemaphysalis aponommoides*, *Haemaphysalis kitaokai* and *Haemaphysalis colasbelcouri* in the territory of China is inconsistent, which may be due to the fact that the identification of species within the subgenus *Alloceraea* is very difficult. As we have not found sound evidence to reject the occurrence of *Ha. inermis* in China, we have kept this species in this dataset.
- (+) *Haemaphysalis kolonini* was reported as a new species in southwestern province Yunnan, China, its morphological and phylogenic characteristics are provided later by Du *et al*.^30^.

Excluded species:

- (−) *Ixodes ricinus*, the most prevalent tick species in Europe^31^, was reported to be found in Fujian, a southeastern coastal province, which is rather distant from its known distribution.
- (−) Five tick species that have been reported only once in pre-2010 publications without providing detailed phenotypic characteristics were considered as needing further confirmation: *Haemaphysalis citelli* and *Ixodes hexagonus* reported in Jilin province in 1962^32^, *Hyalomma aegyptium* reported in Shanxi province in 2005^33^, *Hyalomma marginatum* reported in Gansu in 1987^34^, and *Rhipicephalus annulatus* in Shanxi provinces in 1960^35^.

**Fig. 6:**
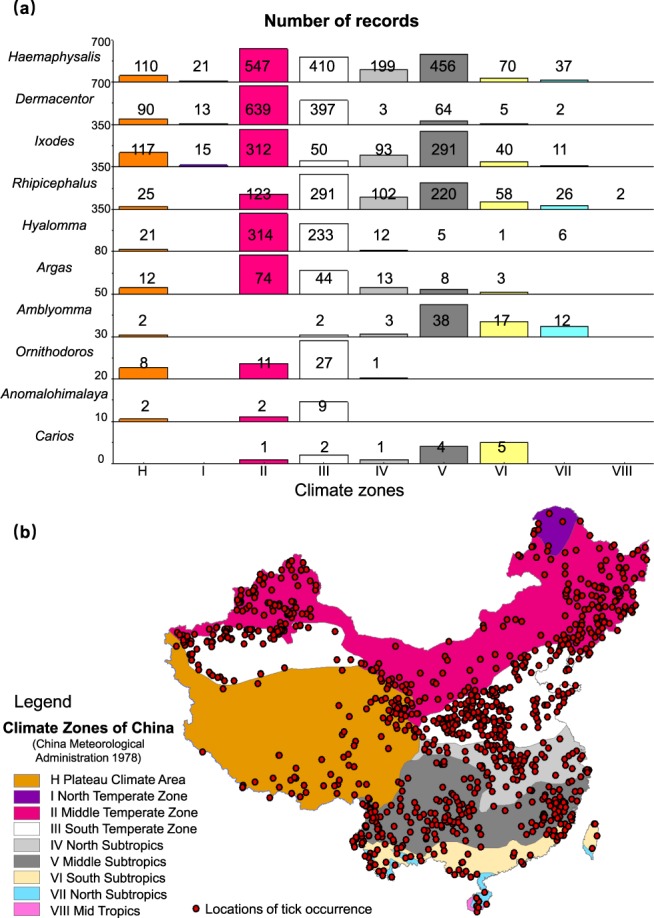
Tick occurrence records in different climate zones. (**a**) Distribution of records of tick genera in different climate zones. (**b**) Geo-locations of tick occurrence on the climate map of China.

The result is a database consisting of 5731 geo-positioned records China for 123 tick species. As of now, ticks are reported to distribute over all the 34 provincial-level divisions in China (Fig. 3), except for Macau Special Administrative Region. Figure 4 shows the diversity and number of records of all the 123 tick species in different provincial-level divisions. We calculated the frequency of report of each tick species across all publications. For each publication, even though a tick species may be reported to be present in different locations, it only counted once. As a result, the six most frequently reported species were identified, and their distributions were mapped in Fig. 5. Though all locations of tick occurrence are recorded as coordinates in the dataset, those on provincial and prefectural levels are displayed as polygons in Fig. 5 for a better visualisation. The distribution of tick occurrences across different climatic zones is displayed and summarised in Fig. 6.

## Usage Notes

Being aware of where disease vectors are present is critical to support policies and direct actions to prevent and manage relevant diseases. Ticks are important transmitter of infectious diseases of global concern. This is the first comprehensive compendium of the distribution of known tick species in China. The dataset described here can be used to investigate the spatio-temporal dynamics of tick distribution at multiple scales. It can also be applied in modelling the ecological risks of tick-borne diseases, in particular in model validation where data are least available^36^.

All the data have been compiled from peer-reviewed literature and error-checked. The dataset has been designed so that potential users (tick ecologist, health geographer, policy maker etc.) can easily filter or aggregate the dataset for their respective investigation purposes and methodologies. Similar datasets of tick distribution in other regions of the world include those for the Western Palearctic region^37,38^, North America^39^, and Africa^40^.

It should be noted the literature reviewed in this study adopted different methods for tick identification, which may introduce a background noise and errors^41^. For example, *Dermacentor reticulatus* and *D. marginatus* exhibit similar phenotypes^42^ and thus difficult to be distinguished previously when genetic evidence was unachievable.

## ISA-Tab metadata file

Download metadata file

## Data Availability

There is no custom code produced during the collection and validation of this dataset.
